# Genetic profile of *GNAQ*-mutated blue melanocytic neoplasms reveals mutations in genes linked to genomic instability and the PI3K pathway

**DOI:** 10.18632/oncotarget.8578

**Published:** 2016-04-04

**Authors:** Mileidys Pérez-Alea, Ana Vivancos, Ginevra Caratú, Judit Matito, Berta Ferrer, Javier Hernandez-Losa, Javier Cortés, Eva Muñoz, Vicente Garcia-Patos, Juan A. Recio

**Affiliations:** ^1^ Biomedical Research in Melanoma-Animal Models and Cancer Laboratory, Oncology Program, Vall d'Hebron Research institute, VHIR-Vall d'Hebron Hospital, Barcelona-UAB 08035, Barcelona, Spain; ^2^ Cancer Genomics Group Translational Research Program, Vall d'Hebron Institute of Oncology-VHIO, Vall d'Hebron Hospital, Barcelona-UAB, Barcelona 08035, Spain; ^3^ Anatomy Pathology Department, Vall d'Hebron Hospital, Barcelona-UAB, Barcelona 08035, Spain; ^4^ Clinical Oncology Program, Vall d'Hebron Institute of Oncology-VHIO, Vall d'Hebron Hospital, Barcelona-UAB, Barcelona 08035, Spain; ^5^ Dermatology Department, Vall d'Hebron Hospital, Barcelona-UAB, Barcelona 08035, Spain

**Keywords:** melanoma, blue nevus, GNAQ, BAP1, genetic profile

## Abstract

Melanomas arising in association with a common or cellular blue nevus (MABN) comprise a relatively rare and heterogeneous group of lethal melanomas. Although *GNAQ* is known to be frequently mutated in common blue nevus, cellular blue nevus (CBN) and MABN and these malignant lesions present gross chromosome alterations harboring *BAP1* mutations, little is known about other mutations that contribute to the development and progression of these neoplasms. Thus, the genetic profile of these tumors is important to increase the number of intervention and treatment modalities. Here, we characterized and genetically profiled two different sections of a rare MABN and two CBNs from three different patients. All of the samples harbored a *GNAQ* mutation, exhibited RAS pathway activation, and harbored additional mutations in genes associated with genomic instability and epigenetic regulation (*KMT2C*, *FANCD2*, *ATR*, *ATRX*, *NBN*, *ERCC2*, *SETD2*, and *WHSC1*). In addition, all neoplasms harbored mutations that directly or indirectly affected either the regulation or activation of the PI3K pathway (*PIK3CA*, *NF1*, *INPP5B* and *GSK3B*). Our results not only help understand the genetic complexity of these blue melanocytic lesions but provide a rationale to use the combination of PI3K/MTOR and MEK1/2 inhibitors against these types of tumors.

## INTRODUCTION

Melanomas associated with blue nevus (MABNs) constitute a class of rare heterogeneous dermal malignant melanocytic lesions that arise in association with common blue nevi (BN), atypical-cellular blue nevi (ACBN) and cellular blue nevi (CBN), [1–4]. The biologic nature of many of these lesions and their malignant potential remain a major problem for histopathologists and clinicians. In this matter, the criteria to differentiate CBN and MABN may not be reliable for many cases, because some cellular blue lesions demonstrate characteristics overlapping those of malignant lesions (MABN). The etiology of MABN is not fully understood, but the presence of longstanding dermal melanocytosis, such as blue nevus, its variants, the nevus of Ota, the nevus of Ito, Mongolian spot, uveal nevus, uveal melanoma, and primary melanocytic neoplasms of the central nervous system, is considered a risk factor [5–7]. By definition, a MABN is a dermal melanoma without the features of melanoma *in situ* that involves the dermo-epidermal junction or adnexal epithelium. It often appears as a deep-seated expansile asymmetric nodule that involves the reticular dermis and subcutaneous fat, although the malignant component may involve the superficial dermis. MABNs are highly aggressive tumors, and effective therapy for the metastatic disease is currently lacking [4].

The genetic changes acquired in primary melanoma are associated with particular anatomical localizations and specific regimes of environmental insults, such as UV radiation [8]. A large proportion of cutaneous melanomas harbor mutations in genes that are part of the mitogen activated protein kinase (MAPK) pathway (i.e., *BRAF* and *NRAS*), which deregulate several important biological processes (proliferation, senescence, survival, and differentiation) in melanocytes. In contrast, *BRAF* mutations are rarely found in uveal melanomas or melanomas arising from the mucosa or internal organs [9–11], and mutations in the receptor tyrosine kinase KIT are found more frequently in lentigo maligna melanoma, acral melanoma and melanomas arising from the mucosa or internal organs [12]. Recent studies have shed new light on the molecular basis of blue nevi [13]. Two members of the Gαq class of G-protein α subunits, which are involved in signaling via G-protein–coupled receptors (GNAQ and GNA11), have emerged as the most important molecules that control early melanoblast proliferation in the dermis [13]. Activating mutations in *GNAQ* and *GNA11* result in a permanent increase in the number of dermal melanoblast [13]. The mutations occur in the RAS-like domain of the protein, leading to a constitutively activated GNAQ protein that essentially converts the GNAQ protein into an activated oncogene product [7]. Somatic mutations in the *GNAQ* gene have been identified in 83% of cases of human blue nevi, 50% of MABNs, and 46% of uveal melanomas [7]. These rates may explain why patients with a nevus of Ota who also harbor *GNAQ* mutations are at a higher risk of developing uveal melanoma [14].

Although the molecular genetics of cutaneous melanomas have been investigated in numerous studies [12, 15, 16], the genetic events that lead to the development of CBN, MABN or ACBN are poorly understood and are limited to a few genes [7, 13, 17–20], including *BAP1* (BRCA1-binding protein 1), an oncogenic deubiquitinase, which has been found to be mutated in metastatic uveal melanomas and MABNs [21]. In this study, we used immunohistochemistry to characterize the melanocyte markers, proliferation markers and relevant pathways involved in melanoma tumor maintenance of two different sections from one MABN and two CBNs. We also genetically profiled the lesions to investigate the mutational status of 386 cancer genes using Haloplex deep sequencing. We then inferred the biological processes that may contribute to malignancy to propose effective therapies.

## RESULTS

### The CBNs and the MABN exhibited histopathological heterogeneity and harbored *GNAQ* mutations

**Figure 1:**
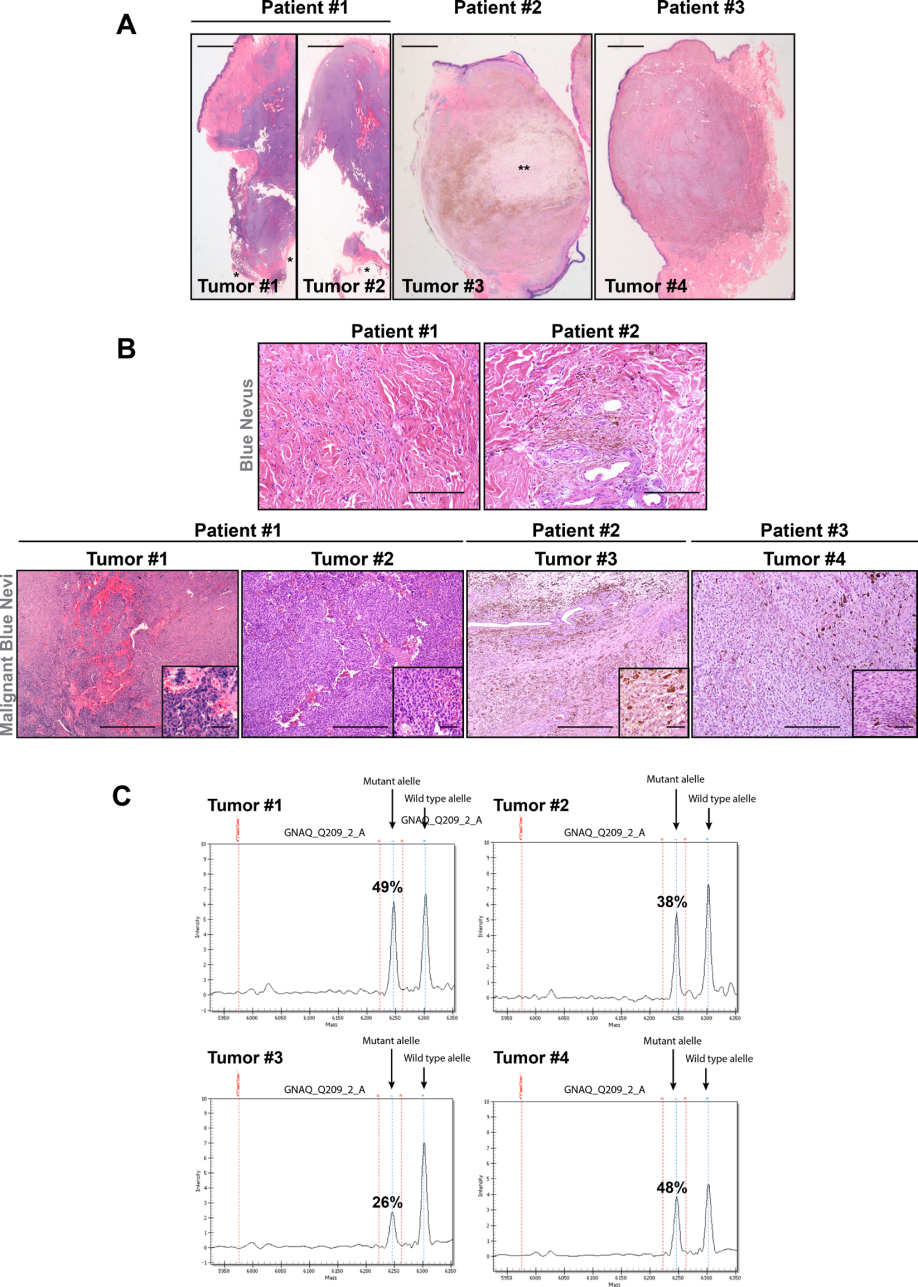
Histological description of *GNAQ*-mutated patient samples **A.** Scanned sections of the tumors (hematoxylin and eosin staining) showing the deep dermal tumor lesions. Four tumors from three different patients were studied. (*) indicates adipose tissue, (**) indicates myxoid area. Bars represent 1 mm. **B.** Upper panels, H&E staining of the nevi associated with the malignant lesions in patient #1 and #2 showing elongated scattered melanocytes. Lower panels show the different histopathological characteristics of all MABN samples. Melanocytes in Tumors #1 and #2 were organized in solid sheets aggregated in nests with hemorrhagic areas. Melanocytes in tumor #3 showed a clear cytoplasm with sparse dusty melanin granules, and tumor #4 showed nests of spindle to oval melanocytes surrounded by collagen and dense fibrous septa. The detailed melanocyte morphology is shown in the magnification of the square area. Bars represent 300 μm and 500 μm in the upper and lower panels, respectively, and 50 μm for insets. **C.** Mass spectrum of the wild-type *GNAQ* and *GNAQ*^c.626A > T^ mutant alleles in tumor samples. Plot denotes the mass height measurement for the two alleles (low mass allele versus high mass allele). The variant allele frequency in each sample is depicted as a percentage.

We initially analyzed the histopathological and molecular characteristics of two different sections from one MABN and two CBNs from three different patients. At scanning magnification, the two sections from the MABN (tumor #1 and #2) were biphasic in appearance and exhibited a variably blue nevus component and distinct cellular areas with an expansive pattern of extension into the subcutaneous adipose tissue (Figure 1A). A cellular blue nevus was present at the superficial and lateral edges of these lesions with small, monomorphous and pigmented melanocytes with prominent dendrites (Figure 1B). The cellular areas were organized in large, solid sheets aggregated in nests with hemorrhagic cleft-like and cystic spaces without an endothelial lining. Melanocytes had a spindle or ovoid appearance and contained little pigment (Figure 1A and 1B). A closer inspection revealed areas of nuclear enlargement with increased nuclei and cytoplasmatic pleomorphism, an increased mitotic rate and the presence of aggregates of melanophages arrayed around nests (Figure 1B). Tumor #3 was an unusual acral CBN that appeared well circumscribed with cystic degeneration in the central part of the lesion (Figure 1A). The tumor cells at the periphery were arranged in nests of spindle to oval melanocytes with inconspicuous nucleoli. The cells exhibited a clear cytoplasm with sparse dusty melanin granules. Numerous melanophages were also present. Neither massive mitosis nor necrosis was detected (Figure 1B). Tumor #4 was a CBN extended vertically into the deep reticular dermis and subcutaneous tissue following adnexal structures or neurovascular bundles. It consisted of multiple nests of spindle to oval melanocytes surrounded by collagen and dense fibrous septa. The cells contained a clear or finely pigmented cytoplasm showing vesicular nuclei with inconspicuous nucleoli. A minimal degree of nuclear pleomorphism or hyperchromasia was observed. The mitotic activity of cells was low (<1 mitosis/mm^2^) (Figure 1A and 1B).

Next, we analyzed 274 mutations in 24 cancer-relevant genes (Sequenom technology), including *BRAF*, *NRAS*, *KIT* c-*MET*, *GNAS* and *GNAQ* (Supplementary Table 1). In agreement with previous results [7], our data showed that all melanocytic blue neoplasms harbored mutations in *GNAQ*^c.626A > T^ at different frequencies (T#1: 49%, T#2: 38%, T#3: 26%, T#4: 48%) (Figure 1C). Thus, the blue melanocytic neoplasms showed heterogeneous histological features and harbored mutations in *GNAQ* which confirmed the diagnosis of these type of lesions.

### The CBNs and the MABNs were positive for c-KIT and exhibited moderate RAS pathway activation

**Figure 2:**
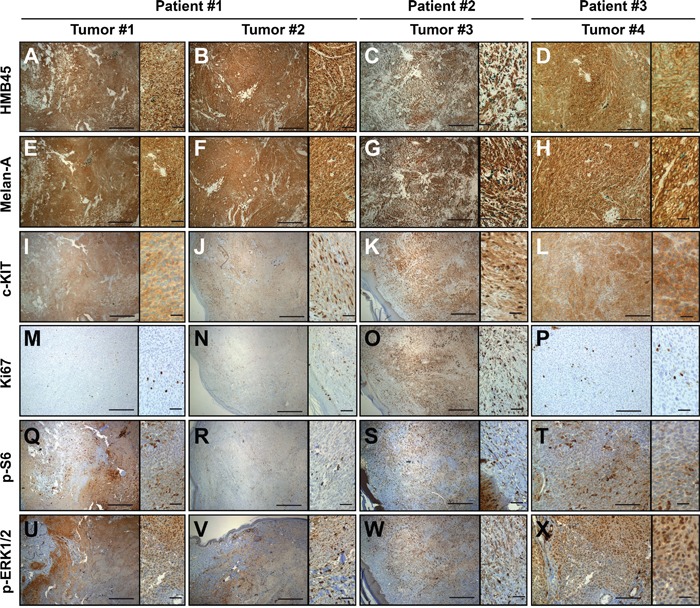
Melanoma and proliferation markers in MABN samples **A-H.** All MABN samples (Tumor #1-#4) were positive for the melanocytic markers HMB45 and Melan-A and the receptor tyrosine kinase c-KIT **I-L.** All tumor samples showed low numbers of Ki67-positive tumor cells **M-P.** Samples showed variable staining for pS6 **Q-T.** and showed positive staining for pERK1/2, mostly at the tumor growth edge **U-X.** The right side of each image shows a magnification from the same image. Bars represent 400 μm, insets represent 50 μm.

Next, we investigated the status of relevant molecules and pathways in melanoma, such as c-KIT and the RAS pathway. All samples were positive for the melanocytic markers HMB45 and Melan-A (Figures 2A–2H) and expressed the receptor tyrosine kinase c-KIT (Figures 2I–2L). According to the proliferation marker Ki67 and concordant with the previous histopathological characteristics (mitotic index), the tumor cell proliferation rate ranged from moderate (MABN) to low (CBNs) (Figures 2M–2P). p-S6 staining, a surrogate marker of mTOR activity, was stronger in tumors #1 and #4 (Figures 2Q–2T) and correlated with the elevated activation of the RAS pathway observed in these tumors. Nevertheless, all tumors, in agreement with their GNAQ mutational status [7], showed an elevated number of p-ERK1/2-positive cells (Figures 2U–2X).

### HaloPlex deep sequencing of CBN and MABN samples reveals genetic alterations that affect genomic instability and cell signaling

**Figure 3:**
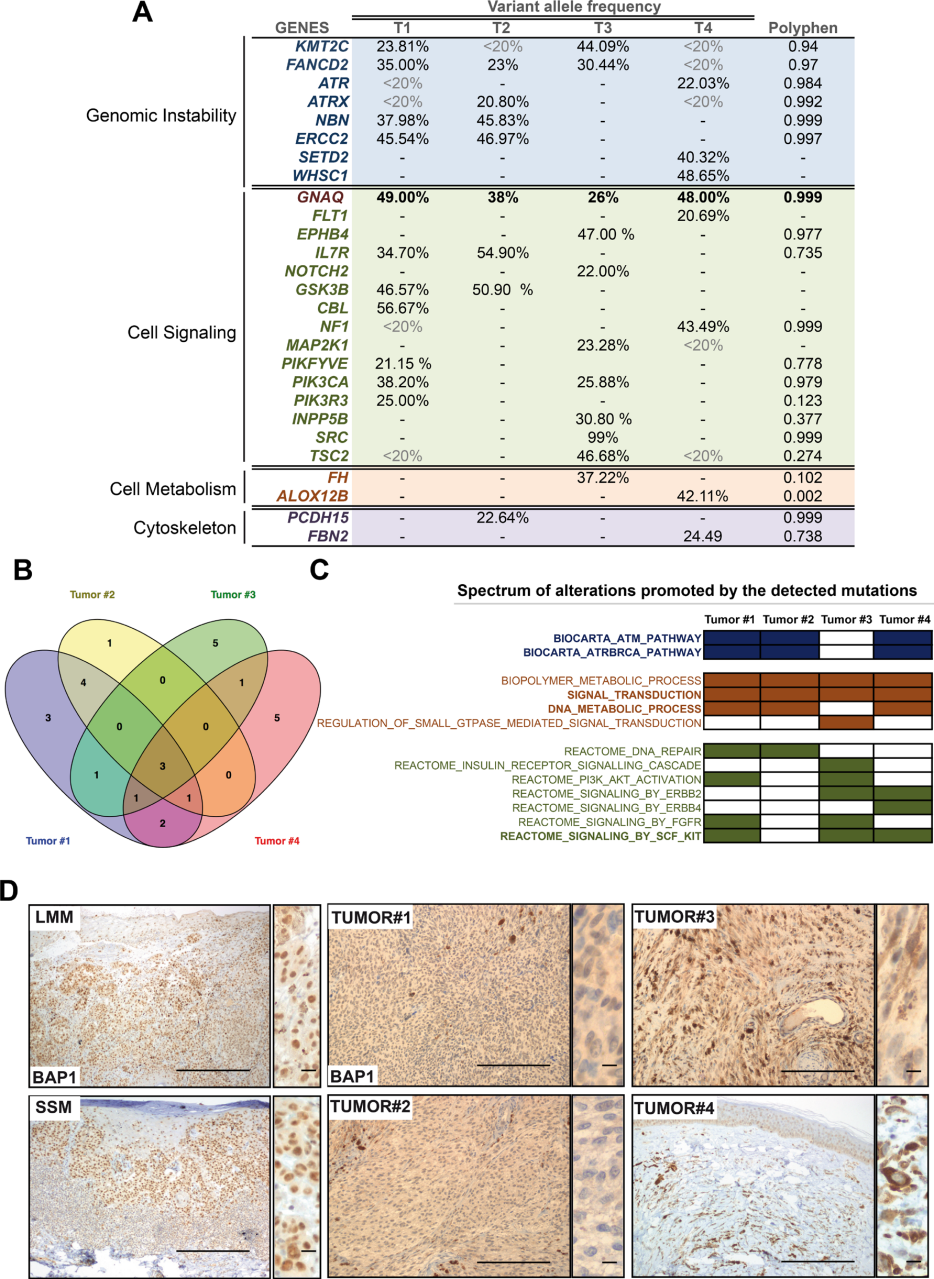
MABN genetic alterations **A.** Genes mutated in tumor samples at a frequency >20%. Mutated genes are arranged according to their biological function. Relevant genes that appear to be mutated in other samples but at a lower frequency (<20%) are shown. **B.** Venn diagram showing common mutated genes observed in (A). **C.** Biological processes altered in the different MABN samples according to a GSEA analysis (http://software.broadinstitute.org/gsea/index.jsp) performed with the mutated gene set from each sample (Biocarta= Blue; Biological Process= Brown; Reactome= Green). **D.** Immunohistochemistry of BAP1 staining in all MABN samples. Normal subcellular localization of BAP1 is shown for Lentigo Maligna Melanoma (LMM) and Superficial Spreading Melanoma (SSM) samples. Magnified images on the right of the pictures show the BAP1 nuclear staining in detail. Bars represent 500 μm (50 μm for the magnified images).

*GNAQ* mutations are reported to occur in 83% of blue nevi [7]. This genetic alteration is also present in other melanocytic lesions, such as uveal melanomas [7] and the Nevus of Ota [22]. Although the genetic mutations that arise during the progression of uveal melanomas and Nevus of Ota are now being clarified with the detection of mutations in *BAP1* and *TP53* [12, 21, 22], the mutational profile of CBNs and rare MABN tumors remains largely unknown. To investigate the genetic profile of these blue melanocytic neoplasms, we performed deep sequencing with a custom Haloplex panel containing 384 cancer-related genes, including *BAP1*, *TP53* and most of the important genes involved in melanoma development and progression (Supplementary Table 2). A total of 37 mutations with an allele frequency higher than 20% were identified in 27 genes among the four tumor samples (32 nonsynonymous substitutions, 4 stop-gains and 1 frame-shift mutation). Among those, C > T; G > A transitions were the most frequent substitutions identified (43%) (Figure 3A, Supplementary Figures 1 and 2). In addition to *GNAQ*, all samples showed mutations in genes related to genomic instability and important signaling pathways (Figure 3A, Supplementary Figure 2 and Supplementary Table 3). The identified mutated genes with high variant frequency that are associated with genomic instability included *KMT2C, FANCD2*, *ATR*, *ATRX*, *NBN*, *ERCC2*, *SETD2*, and *WHSC1* (Figure 3A and Supplementary Table 3). Interestingly, in addition to the *GNAQ*^c.626A > T^ mutation, the four blue melanocytic neoplasms showed changes in important genes that activate the RAS and PI3K signaling pathways (Figure 3A and Supplementary Table 3). Tumor #1 and Tumor #2, the two different sections that belong to the same patient, showed mutations in *GSK3β* (at a variant allele frequency of approximately 50%). Tumor #1, in addition to the mutations observed in Tumor #2, also harbored mutations in *CBL* (56.7%), *PIKFIVE* (21%), *PIK3CA* (38.2%), and *PIK3R3* (25%), (Figure 3A). Mutations in *PIK3CA* (25.9%), *INPP5B* (30.8%), *SRC* (99%) and *TSC2* (46.7%) were observed in Tumor #3, and Tumor #4 harbored an *NF1* gene mutation (43.5%). Though they occurred at different frequencies, three genes were found mutated in all tumor samples: *GNAQ*, the histone methyltransferase *KMT2C*, and the Fanconi anemia group D2 protein *FANCD2*; the latter two are involved in epigenetic transcriptional activation and the maintenance of chromosomal stability, respectively [23–25] (Figure 3B). TSC2 was mutated in three of four samples, although with very low frequency (Figure 3B). According to the TCGA database (cBioportal for cancer genomics; http://www.cbioportal.org), these genes all appear to be altered to different extents in cutaneous and desmoplastic melanoma (278 cases and 20 cases, respectively), whereas *NBN* appears to be altered in 21% of uveal melanoma cases (80 cases) (Supplementary Figure 3). A gene set enrichment analysis (http://software.broadinstitute.org/gsea/login.jsp) of the mutated genes (only genes with a frequency of >20%) for each sample confirmed that the main processes altered in these tumor cells were related to genomic instability and the deregulation of different mechanisms that are relevant to several signaling pathways, such as the RAS, PI3K and c-KIT pathways (Figure 3C). These data were reinforced when we analyzed the known and predicted protein-protein interactions networks of the identified mutated genes (Supplementary Figure 4).

A number of histological and genetic alterations (*GNAQ* mutation) [7, 14, 22] overlap among uveal melanoma, melanoma associated with a nevus of Ota and melanoma associated with a blue nevus, suggesting that tumor-initiating mutations and their pathway requirements determine subsequent genetic alterations during progression [22]. *BAP1* has been found to be frequently mutated in uveal melanoma [21] and in one case of melanoma associated with a nevus of Ota [22]. Our results showed that the four tumor samples harbored a wild-type BAP1 allele, but all samples either expressed a cytoplasmic delocalized BAP1 or did not express the protein (Figure 3D), suggesting a convergent inactivation mechanism of BAP1 in GNAQ-mutated samples. In addition to this, all the samples showed a negative staining for p53 (Supplementary Figure 5). Overall, these data suggest that these blue melanocytic neoplasms developed and progressed increasing their genomic instability and deregulating the RAS and PI3K pathways.

## DISCUSSION

In cutaneous melanomas, alterations in the RAS pathway (BRAF, NRAS and NF1) are important for melanoma development and tumor maintenance [26], whereas the Gαq class of G-protein α subunits GNAQ and GNA11 seem to be important founder molecules in uveal melanoma and other dermal lesions, such as blue nevus, nevus of Ota and nevus of Ito [5, 7, 22]. A number of genes are involved in the progression of cutaneous melanomas [26], but *BAP1* is the only prevalent gene associated with malignant progression in uveal melanoma, melanomas associated with a nevus of Ota and MABN [21, 22]. Here, we characterized and genetically profiled four blue melanocytic neoplasms from three different patients and analyzed 384 cancer-related genes using HaloPlex deep sequencing to better understand the changes associated with the progression of these rare lesions.

In agreement with previous data illustrating that 83% of BNs and 50% of MABNs harbored *GNAQ* mutations [7], *GNAQ* was mutated in all of our tumor samples at a high frequency, thus confirming that GNAQ mutation is an initiating event. Interestingly, the genetic profile of our samples revealed two main categories of mutated genes that affect either genomic instability or relevant melanoma signaling pathways. Among the mutated proteins that contribute to the genomic instability, FANCD2 plays a role in preventing the breakage and loss of mis-segregating chromatin at the end of cell division, by interacting with BRCA1 [28] and BRCA2 [24] and converging with the ATR signaling pathway [23]; Nibrin (NBN) is a component of the MRE11-RAD50-NBN (MRN complex), which plays a critical role in the cellular response to DNA damage and the maintenance of chromosome integrity [29, 30]. Furthermore, ERCC2 (XPD) is involved in nucleotide excision repair (NER) and chromosome segregation, which alterations have been associated with an increased risk of skin cancer [31]. According to the TGCA database, all these genes are involved in melanoma (Supplementary Figure 3); where NBN is particularly altered in uveal melanoma. We do not know the direct effect of these mutations on tumor progression (genomic instability). However, the elevated number of chromosome aberrations observed in these types of samples [27, 32] warrants the investigation of the mutational status of these genes in a larger set of samples.

The progression and metastasis of uveal melanoma and MABNs have been associated with a loss of function of BAP1 [21, 27], which is located on chromosome 3. BAP1 is a tumor suppressor that is believed to modulate chromatin, regulate transcription, and possibly affect the ubiquitin-proteasome system and the DNA damage response pathway. Several studies have shown that MABNs harbor a number of gross chromosome losses and chromosome rearrangements, including the partial deletion of the *BAP1* locus (3p2.1), evidencing the elevated genomic instability in these tumors [27]. *BAP1* was not mutated in our set of samples, but it was either delocalized in the cytoplasm or absent, thus suggesting the functional inactivation of BAP1. Although we do not have a defined mechanism(s) for this observation, several possibilities are conceivable. BAP1 suffers post-translational modifications, that regulate its interaction with other proteins and substrates (phosphorylation) [Eletr, 2013 #166][Okino, 2015 #167], and its subcellular localization (ubiquitination) [Mashtalir, 2014 #168]. In this matter, the regulation of its ubiquitination and the activity of the kinases responsible for BAP1 phosphorylation are not well defined and could be altered in the tumoral context. Moreover, because BAP1 interacts with BRCA1/2 proteins, the contribution of these mutations to BAP1 functional inactivation (i.e., shuttling between nucleus and cytoplasm) should also be investigated. Additionally, the copy number variation of the BAP1 locus could be responsible for the lack of BAP1 expression [27, 32]. However, Tumors #3 and #4 harbored mutations in the histone methyltransferases KMT2C, SETD2 and WHSC1, which may directly contribute to the epigenetic transcriptional regulation [33] of *BAP1*. Considering that *BAP1* mutational status correlates with malignancy, and BAP1 appear to be functionally inactivated in CBNs, it could be inferred that these type of lesions represent a spectrum (benign to malignant) of blue melanocytic neoplasms very difficult to classify according to their histological features, which denotes the necessity of a stricter control of these patients.

With respect to the signaling pathways altered in these tumors, mutations in *GNAQ* and *GNA11* appear to activate the RAS pathway in MABN, uveal melanoma and other melanocytic lesions [7]. Our set of samples harbored mutations at *GNAQ*^c.626A > T^; consequently, all tumors were positive for p-ERK1/2. Interestingly, the mutational analysis of samples and the effect of these alterations on the signaling pathways (S6 phosphorylation) indicated that the PI3K pathway was directly or indirectly activated in all samples. Multiple genetic and epigenetic aberrations that activate this pathway have been identified *de novo* and in acquired resistance melanoma models [34]. In our set of samples, Tumor #1 and Tumor #3 carried mutations in *PIK3CA*, with variant allele frequencies of 38% and 26%, and Tumor #4 harbored an *NF1* mutation, which might activate the PI3K signaling pathway. Furthermore, the genetic profile of the two different sections from Patient #1 (Tumors #1 and #2) indicate that Tumor #1 may have clonally evolved from Tumor #2 by acquiring mutations in the PI3K pathway, which supports the relevance of the mutations in this pathway during the progression of this malignancy. Interestingly, this patient was also positive for sentinel lymph node. Intriguingly, a *SRC* mutation (99% frequency, probably copy-neutral LOH), which is a very infrequent mutation in melanoma, was detected in Tumor #3, which was a rare acral CBNs with a cystic degeneration in the central part of the tumor.

The use of MEK1/2 inhibitors has been suggested as a therapy for *GNAQ*-mutated uveal melanomas. Moreover, the combination of MEK1/2 inhibitors with dual PI3K/MTOR inhibitors, or PKC inhibitors, enhanced uveal melanoma cell death in a mutant *GNAQ*- and *GNA11*-dependent manner [35, 36]. Accordingly, our results suggest that activation of RAS pathway and PI3K pathway might be involved in the development and progression of this type of lesions. Thus, these therapeutic approaches might also be effective against some malignant blue melanocytic tumors.

In summary, we herein studied the genetic profile and pathway alterations of four different *GNAQ*-mutated blue melanocytic neoplasms from three different patients. The results showed that these tumors harbor mutations in genes related to genomic instability, which correlates with the elevated number of chromosomal aberrations observed in this type of tumor. Importantly, the data also show that in addition to the *GNAQ*-mediated activation of the RAS pathway, PI3K pathway activation is likely required for the development and progression of these tumors. These results establish a rationale for the combined use of PI3K/MTOR and MEK1/2 inhibitors in the treatment of these rare and lethal lesions.

## MATERIALS AND METHODS

### Patients

Four samples of melanoma associated with a blue nevus from three different patients were provided by the Tumor Bank of the Vall d'Hebron University Hospital Biobank under the appropriate ethical approval (supported by the Catalonia Tumor Bank Network) sponsored by Pla Director d'Oncología de Catalunya (XBTC); supported by Platform Biobank (ISCIII).

### Immunohistochemistry and immunofluorescence

Formalin-fixed paraffin embedded (FFPE) tumor samples were subjected to immunocytochemistry according to the manufacturer's antibody protocol. The samples were developed using secondary antibodies conjugated with horseradish peroxidase and diaminobezidine as a substrate. Immunohistochemical staining was performed on 4 μm sections from formalin-fixed paraffin-embedded tissues. Melan-A, HMB-45 and Ki67 were obtained from Master Diagnostica (Granada, Spain); c-KIT, p-S6 and p-ERK1/2 were purchased from Cell Signaling (Danvers, MA, USA). The paraffin sections were automatically de-paraffinized and treated with cell conditioning 1 solution (pH8) for antigen retrieval (Ventana Medical Systems, Tucson, AZ, USA). Staining was performed with an automated immunostainer (Beckmarck XT, Ventana Medical Systems). Antibodies were visualized using the ultraViewTM Universal DAB detection Kit (Ventana Medical Systems). The samples were evaluated by two independent pathologists.

### DNA extraction

The tumor area content of FFPE samples was evaluated by two independent pathologists. Whenever possible, samples were macro-dissected to enrich for nevus/tumor content. DNA was extracted from FFPE tumor samples using the Maxwell® 16 FFPE Plus LEV DNA Purification Kit.

### Sequenom oncogene mutation profiling

The MassARRAY system (Sequenom, CD Genomics, Shirley, NY, USA) and two assay panels, OncoCarta v1.0 and CLIA v2.2, were used to somatically profile 24 oncogenes (see Supplementary Table 1). The panels contain a total of 273 assays based on IPlex chemistry (Sequenom). DNA was extracted from 5×10 mm slices of FFPE tumor samples using the Maxwell® 16 FFPE Plus LEV DNA Purification Kit. Briefly, 600 ng of DNA was used for mutation profiling with OncoCarta v1.0, and 180 ng was used for the CLIA v2.2 panel. Briefly, after quantification (Nanodrop, Thermo-Fisher, Walthman, MA, USA) and the dilution of DNA to a 10 ng/ml, multiplexed PCR was performed to amplify the genomic regions that contain the loci to be genotyped (5/ml volumes containing 0.1 units of Taq-polymerase, 20 ng of genomic DNA, 2.5 pmol of each PCR primer and 2.5 pmol of dNTP. The following thermocycling conditions were employed: 95°C for 15 min followed by 45 cycles of 95°C for 20 s, 56°C for 30 s and 72°C for 30 s). Unincorporated dNTPs were deactivated by the addition of shrimp alkaline phosphatase (0.3 U) and incubation at 37°C for 40 min, followed by the heat inactivation of the enzyme for 5 min at 85°C. Subsequently, each mutation was analyzed as the single-base extension product of a probe that annealed immediately contiguous to the mutation position. Primer extension was carried out by adding 5.4 pmol of each primer extension probe, 50 mmol of ddNTP and 0.5 units of Thermosequenase DNA polymerase to the amplification products, which were then incubated at 50°C for 5 s and 72°C for 5 s. After the addition of a cation exchange resin to remove residual salt from the reactions, 7 nl of the purified primer extension reaction was loaded onto a matrix pad (3-hydroxypicoloinic acid) of a Gen II SpectroCHIP (Sequenom). Gen II SpectroCHIPs were loaded into a matrix-assisted laser desorption/ionization–time of flight (MALDI-TOF) mass spectrometer (Mass ARRAY, Sequenom), and spectra were obtained for each of the extension products. Data analysis and mutation reports were generated using the Typer Analyzer 4.0 software (Sequenom). The spectrum of each of the reported mutations was manually assessed using the Sequenom software (MAF 10%).

### HaloPlex sequencing

A custom HaloPlex panel covering 1.4 Mbp in 384 genes was used to profile the blue nevus-associated melanoma samples (Supplementary Table 2). The resulting libraries were loaded on a HiSeq2000 (Illumina, San Diego, CA, USA) instrument 2X100 and sequenced to an average coverage of 800X. Blue nevus melanoma-associated samples were filtered for common SNPs according to their control (Blood) and the 1000 genome SNP catalogue. The resulting missense variants were prioritized based on their previous detection in tumors (COSMIC database, TCGA), the predicted impact on protein function (Polyphen-2, SIFT), and the location in relevant protein domains in these genes.

## SUPPLEMENTARY FIGURES AND TABLES


