# Supplementary material for: Genetic profile of GNAQ-mutated blue melanocytic neoplasms reveals mutations in genes linked to genomic instability and the PI3K pathway
Source: Oncotarget. 2016 Apr 4;7(19):28086–95. doi: 10.18632/oncotarget.8578 (PMC5053711; doi:10.18632/oncotarget.8578)
Supplement: Supplementary file 1 [file oncotarget-07-28086-s001.pdf]

SUPPLEMENTARY FIGURES AND TABLES

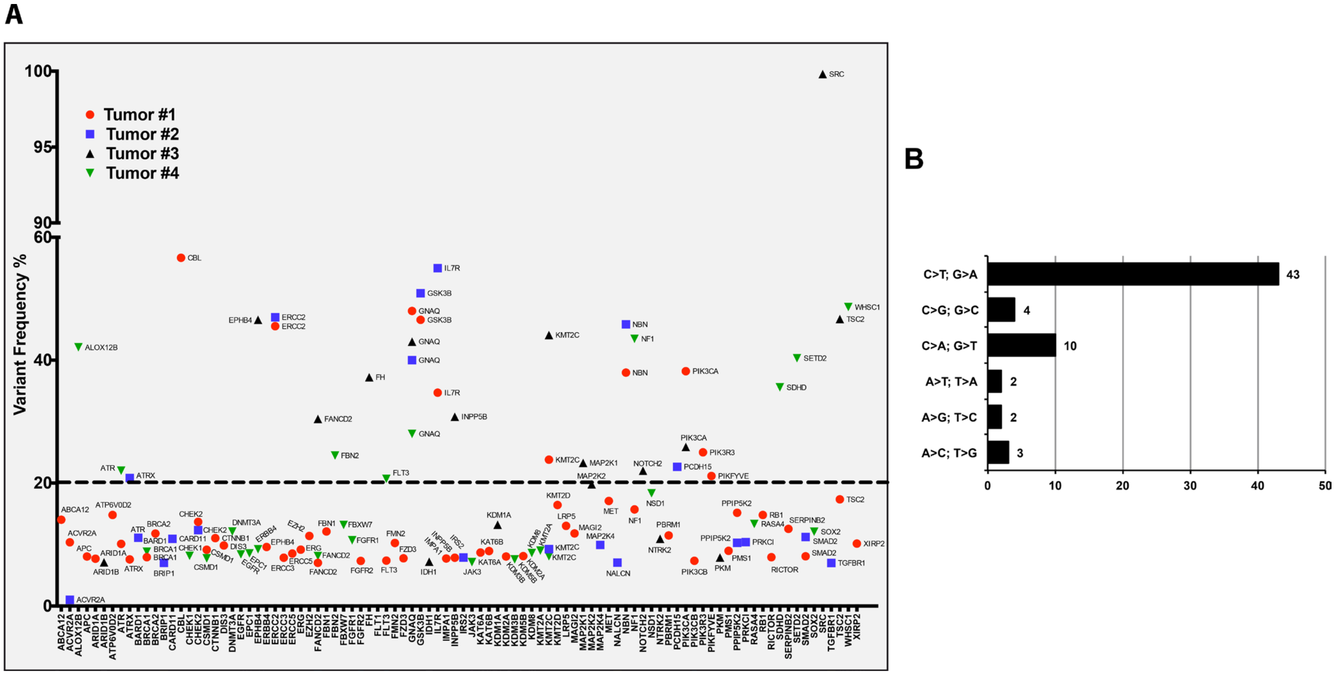

Supplementary Figure S1: Somatic mutations identified by HaloPlex followed of deep-sequencing. A. Graph showing the mutated genes and the variant allele frequency in percentage. **B.** Graph shows the percentages of the type of base substitution found among all samples.

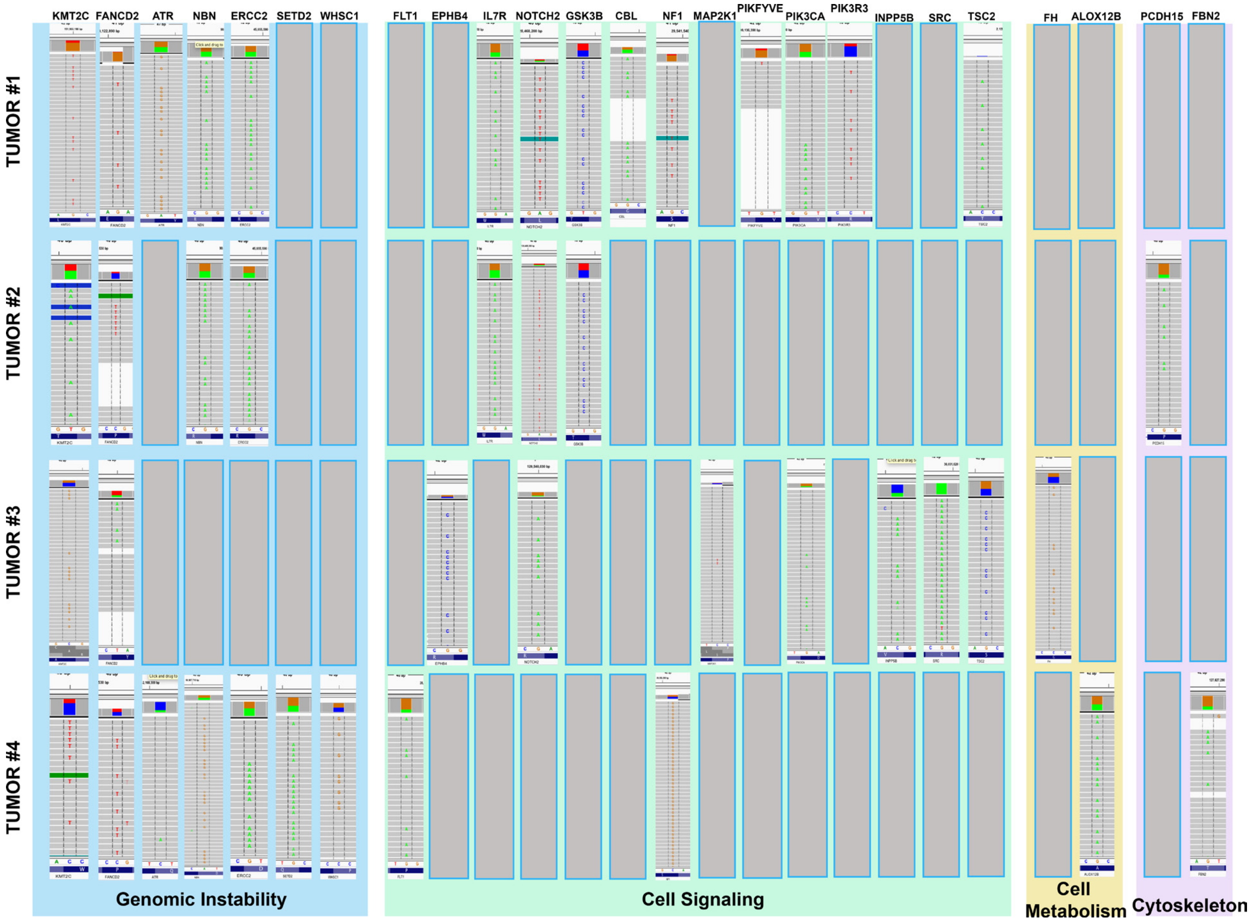

Supplementary Figure S2: Images of the IGV genome browser view of the reads from the non-synonymous mutations identified by deep sequencing. Genes are grouped according the biological process they are involved in. Blue: genomic instability, Green: Cell signaling, Yellow: cell metabolism, Purple: Cytoskeleton. Grey rectangle: wild type gene.

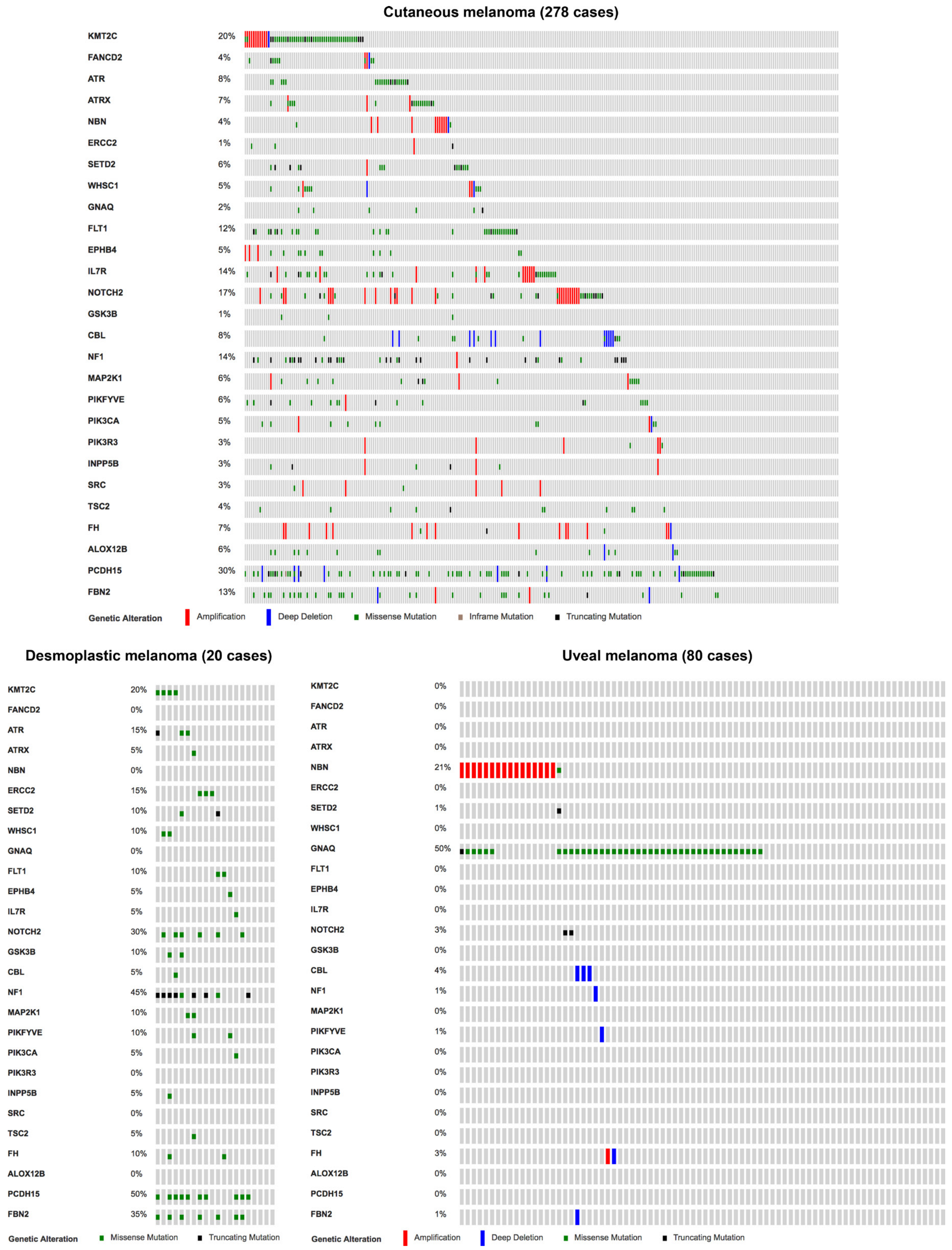

Supplementary Figure S3: Identified mutated genes are also mutated in cutaneous melanoma desmoplastic melanoma and uveal melanoma. Mutated genes identified in MABN samples at a frequency >20% were analyzed using the TCGA database (cBioportal for cancer genomics; http://www.cbioportal.org). Results for the different melanoma subtypes and the number of cases in each group are shown.

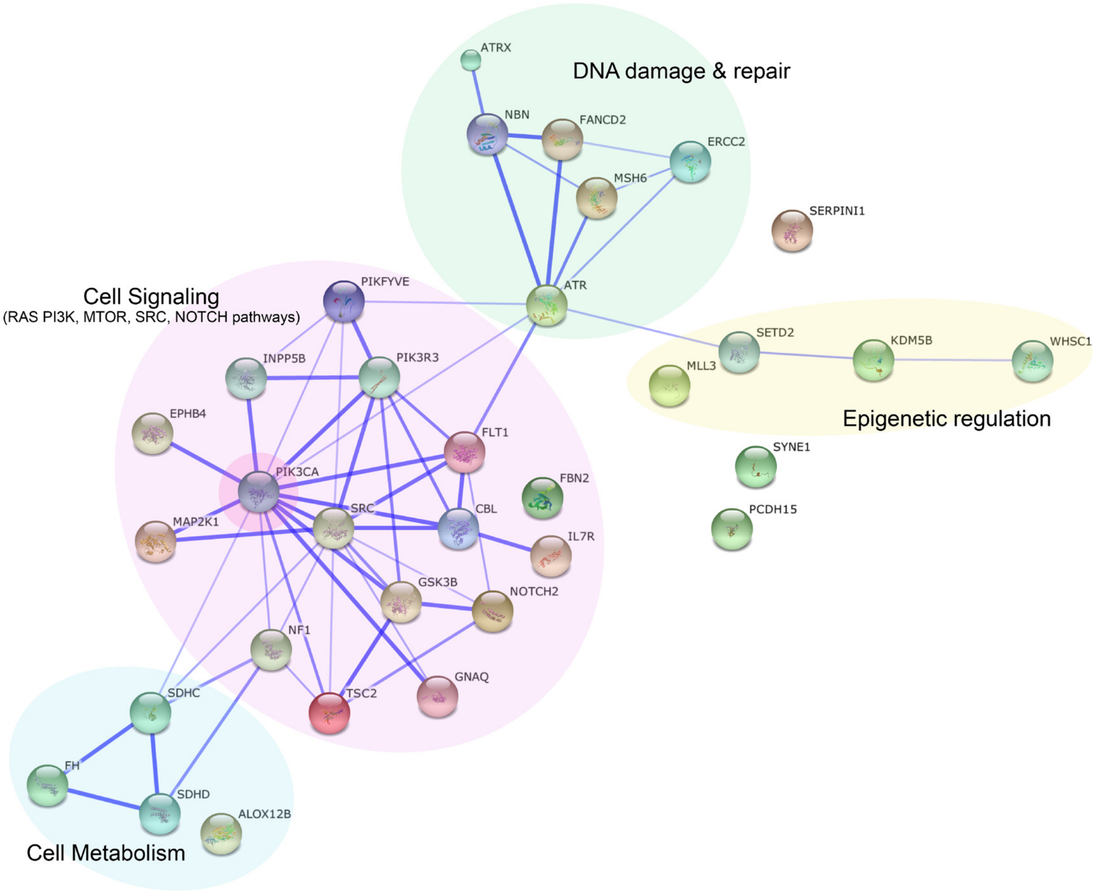

Supplementary Figure S4: Known and predicted protein-protein interactions. Association among the mutated genes detected in melanoma associated to blue nevus samples (STRING, Search Tool for the Retrieval of Interacting Genes/Proteins, http://string-db.org/newstring_cgi). This is the confidence view. Stronger associations are represented by thicker lines.

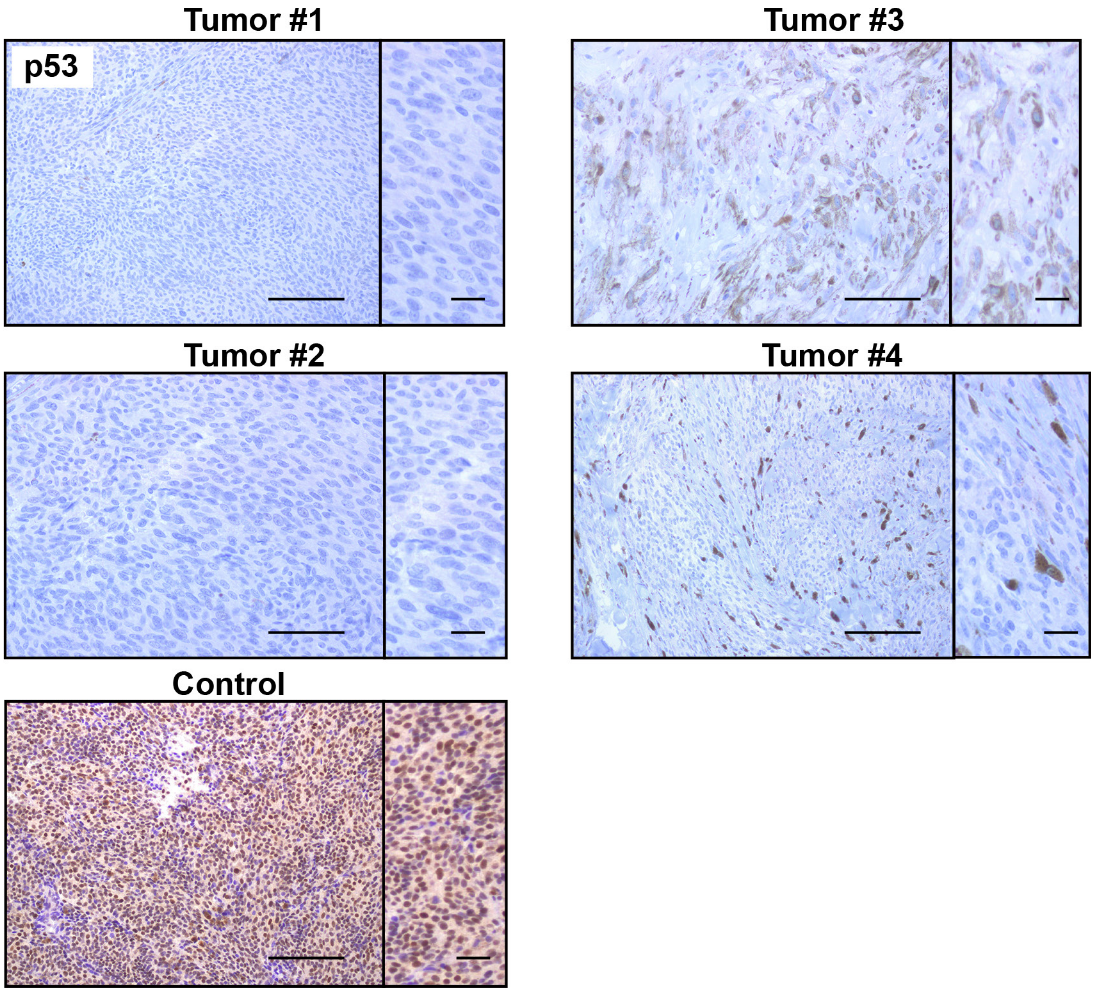

Supplementary Figure S5: Tumors samples were stained with p53 antibody. A tumor control for the staining is showed.

Supplementary Table S1: List of oncogene mutations evaluated by Sequenom technology

ONCOGENE MUTATIONS								
Gene	Mutation							
ABL1	G250E	F317L	M351T	E355G	F359V	H396R	Q252H	Y253H
	Y253F	E255K	E255V	D276G	F311L	T315I		
AKT1	rs11555435	rs11555431	rs11555432	rs12881616	rs11555433	rs11555436	rs34409589	E17K
	G173R	K179M						
AKT2	S302G	R371H	E17K	G175R				
AKT3	E17K	G171R						
BRAF	G464R	F595L	G596R	L597S	L597R	L597Q	L597V	T599I
	V600E	V600K	V600R	V600L	K601N	K601E	G464V	G464E
	G466R	F468C	G469S	G469E	G469A	G469V	G469R	G469R
	D594V|G							
CDK	R24C	R24H						
EGFR	R108K	S768I	V769_D770 insASV	V769_D770 insCV	D770_N771 >AGG	V769_D770 insASV	V769_D770 insASV	D770_N77 1insG
	N771_P772 >SVDNR	P772_H773 insV	H773>NPY	H773_V774 insNPH	H773_V774 insPH	H773_V774 insH	V774_C775 insHV	T263P
	T790M	L858R	L861Q	A289V	G598V	E709K	E709H	E709A
	E709G	E709V	G719S	G719C	G719A	M766_A767 insAI	E746_T751 del	E746_A750 del
	E746_A750 del	E746_T751 del	E746_A750 del	E746_T751 del, S752D	L747_E749 del	L747_T750 del	L747_S752 del	L747_T751 del
	L747_S752 del, P753S	L747_T751 del	A750P	T751A	T751P	T751I	S752I/F	L747_Q ins
	E746_T751 del, I ins	E746_A750 del, T751A	E746_T751 del, V ins	E746_A750 del, V ins	L747_E749 del, A750P	L747_T750 del, P ins	L747_S752 del, Q ins	T751
	S752_I759d el							
ERBB2	L755P	G776S	G776LC	G776VC	A775_G776 insYVMA	P780_Y781 insGSP	P780_Y781 insGSP	S779_P780 insVGS
	G309A	H470Q	I767M	R678Q	V842I			
FGFR1	S125L							
FGFR3	P252T	G370C	Y373C	A391E	K650Q	K650E	K650T	K650M
FLT3	I836del	D835H	D835Y					
HRAS	G12V	G12D	G13C	G13R	G13S	Q61H	Q61L	Q61R
	Q61P	Q61K	A146T	A146P	A146V			
KRAS	G12A	G12V	G12D	G12S	G13C	G13S	G13D	Q61E
	Q61H	Q61K	Q61L	Q61P	Q61R			
NRAS	G12V	G12A	G12D	G12C	G12R	G12S	G13V	G13A
	G13D	G13C	G13R	G13S	A18T	Q61L	Q61E	Q61H
	Q61K	Q61P	Q61R					
IDH1	R132C	R132G	R132H	R132L	R132S	R132V		
IDH2	R172G	R172K	R172M	R172S	R172W			
JAK2	V617F							
KIT	D52N	V559del	V559_V560 del	V560del	P551_V555 del	Y553_Q556 del	Y570_L576 del	E561K
	L576P	P585P	D579del	Y503_F504i nsAY	K642E	D816V	D816H	D816Y
	V825A	E839K	M552L	Y568D	F584S	W557R	W557G	V559D
	V559A	V559G	V559I	V560D	V560G	K550_K558 del	K558_V560 del	K558_E562 del
MET	R970C	T992I	Y1230C	Y1235D	M1250T	H1112L	H1112R	H1112Y
	H1124D	M1268T	N375S	N848S	R988C	T1010I	Y1248C	Y1248H
PDGFRA	V561D	I843_S847> T	D842V	T674I	F808L	D846Y	N870S	D1071N
	D842_H845 del	I843_D846 del	S566_E571 >K					
PIK3CA	R88Q	H1047Y	R38H	C901F	M1043I	M1043I	N345K	C420R
	P539R	E542K	E545K	Q546K	H701P	H1047R	H1047L	H1047Y
	A1046V	C420R	E110K	E418K	E453K	E542Q	E542V	E542G
	E545A	E545D	E545G	E545Q	E545V	F909L	K111N	M1043I
	M1043V	Q60K	Q546E	Q546H	Q546L	Q546P	Q546R	S405F
	T1025A	T1025S	Y1021C	Y1021H	G1049R	G1049S		
GNAQ	Q209H	Q209L	Q209P	Q209R	Q209Y			
GNAS	Q227L	Q227R	R201H					
RET	C634R	C634W	C634Y	E632_L633 del	M918T	A664D		

Supplementary Table S2: Genes investigated by Haloplex exon sequencing

Genes in Haloplex panel:

ABCA12	BRIP1	DKK2	FAM46C	HNF1A	LGR6	NCOA2	PKM2	SERPINB1	TMPRSS2
ABCA7	BUB1B	DKK3	FANCA	HRAS	LRP1B	NEB	PLK2	SERPINB2	TNFAIP3
ABL1	CARD11	DKK4	FANCC	HSP90AA1	LRP5	NF1	PLK3	SERPINB3	TNFRSF14
ABL2	CASP8	DMD	FANCD2	IDH1	MAGI2	NF2	PMS1	SERPINB4	TOP1
ACVR1B	CBL	DNAH5	FANCE	IDH2	MAP2K1	NFE2L2	PMS2	SERPINB5	TP53
ACVR2A	CBLB	DNM2	FANCF	IGF1R	MAP2K2	NFKB1	PNRC1	SERPINE1	TP63
ADAMTS20	CBLC	DNMT1	FANCG	IGFBP7	MAP2K4	NFKB2	POLE	SERPINI1	TSC1
AFF2	CCND1	DNMT3A	FAS	IKBKE	MAP3K5	NKX2-1	PPP2R1A	SERPINI2	TSC2
AKT1	CCNE1	DNMT3B	FBN1	IKZF1	MAP3K8	NOTCH1	PRDM1	SETD2	TSHR
AKT2	CD79B	DOCK2	FBN2	IL7R	MAP3K9	NOTCH2	PREX2	SF3B1	U2AF1
AKT3	CDC27	DPP6	FBXO11	INSR	MAP7	NOTCH3	PRKAR1A	SHQ1	VHL
ALK	CDC42EP2	ECT2L	FBXW7	IRS1	MAPK12	NOTCH4	PRKCI	SKI	WAS
ALOX12B	CDC73	EDNRB	FGFR1	IRS2	MCL1	NPM1	PTCH1	SKIL	WBSCR17
APC	CDH1	EGFR	FGFR2	JAK1	MDM2	NRAS	PTEN	SLC16A4	WHSC1
AR	CDH10	EIF4EBP1	FGFR3	JAK2	MDM4	NSD1	PTPN11	SLC9A9	WHSC1L1
ARAF	CDK12	EP300	FGFR4	JAK3	MEN1	NTRK1	PTPN12	SMAD2	WRN
ARHGAP26	CDK4	EPC1	FH	JUN	MET	NTRK2	PTPRD	SMAD3	WT1
ARID1A	CDK5	EPHA3	FLCN	KAT6A	MIER3	NTRK3	PTPRS	SMAD4	XIRP2
ARID1B	CDK6	EPHA5	FLT1	KAT6B	MITF	OR10R2	PXDN	SMAD7	XPA
ARID2	CDK8	EPHA6	FLT3	KDM1A	MLH1	PAK7	RAD51	SMARCA4	XPC
ASXL1	CDKN2A	EPHA7	FMN2	KDM2A	MLH3	PALB2	RAF1	SMARCB1	XPO1
ATM	CDKN2B	EPHA8	FOXL2	KDM2B	MLL	PARK2	RARA	SMO	YAP1
ATP6V0D2	CDKN2C	EPHB1	FUBP1	KDM3B	MLL2	PARP1	RASA1	SOCS1	YES1
ATR	CEBPA	EPHB4	FZD3	KDM4A	MLL3	PAX5	RASA2	SOX2	ZIM2
ATRX	CHEK1	EPHB6	GATA1	KDM4B	MLST8	PBRM1	RASA3	SPOP	ZNRF3
AURKA	CHEK2	ERBB2	GATA2	KDM4C	MPL	PCDH15	RASA4	SRC	ZRSR2
AXIN1	CIC	ERBB3	GATA3	KDM5A	MSH2	PDGFRA	RB1	SRSF2	
AXIN2	CREBBP	ERBB4	GNA11	KDM5B	MSH3	PDGFRB	RECQL4	STK11	
BAI3	CRKL	ERCC2	GNAQ	KDM5C	MSH6	PDZRN3	REL	SUFU	
BAP1	CRLF2	ERCC3	GNAS	KDM6A	MTOR	PHF6	RET	SYNE1	
BARD1	CSF1R	ERCC4	GOLPH3	KDM6B	MUC16	PHOX2B	RICTOR	TBK1	
BCL2L1	CSMD1	ERCC5	GPC3	KDM8	MUTYH	PIK3C2G	RNF43	TCERG1	
BCL6	CTNNA2	ERG	GPC6	KDR	MYB	PIK3CA	RPTOR	TCF7L2	
BCOR	CTNNB1	ESR1	GRIK3	KEAP1	MYC	PIK3CB	RUNX1	TEK	
BIRC2	CYLD	ETV1	GRIN2A	KIT	MYCL1	PIK3CD	SBDS	TERT	
BLM	DAXX	ETV6	GSK3B	KLF6	MYCN	PIK3CG	SCN5A	TET1	
BMPR1A	DDR2	EXT1	H3F3A	KLHDC4	MYD88	PIK3R1	SDHB	TET2	
BRAF	DICER1	EXT2	HDAC2	KRAS	MYO1B	PIK3R2	SDHC	TET3	
BRCA1	DIS3	EZH2	HIF1A	LDHA	NALCN	PIK3R3	SDHD	TGFBR1	
BRCA2	DKK1	FAM123B	HMGA2	LEFTY1	NBN	PIM1	SERPINA9	TGFBR2	

Supplementary Table S3: List of genes mutated identified by haloplex-exon sequencing in blue melanocytic neoplasms

	Freq.	Freq.	Freq.	Freq.	Nucl. Ref	Nucl. Var	Polyphen
ABCA12	14.04%	-	-	-	G	A	0.032
ACVR2A	10.37%	6%	-	-	G	A	1
ALOX12B	-	-	-	42.11%	G	A	0.02
APC	8.05%	-	-	-			
ARID1A	7.69%	-	-	-			
ARID1B	-	-	7.10%	-	C	T	0.948
ATP6V0D	14.81%	-	-	-	C	A	0.211
ATR	10.10%	-	-	22.03%	C	A	0.984
ATRX	7.59%	20.83%	-	-	C	A	0.803
BARD1	-	11.11%	-	-	G	T	0.014
BRCA1	7.94%	-	-	8.84%			
BRCA2	11.79%	-	-	-			
BRIP1	-	7.02%	-	-	C	T	0.001
CARD11	-	10.93%	-	-	G	A	0.999
CBL	56.67%	-	-	-	G	A	0.763
CHEK1	-	-	-	8.18%	G	A	0.011
CHEK2	13.70%	12.35%	-	-	T	G	0.032
CSMD1	9.16%	-	-	7.77%			
CTNNB1	11.04%	-	-	-	C	T	0.996
DIS3	9.85%	-	-	-			
DNMT3A	-	-	-	12.12%	G	A	0.999
EGFR	-	-	-	8.40%	A	G	0.999
EPC1	-	-	-	8.56%	G	T	0.035
EPHB4	-	-	46.56%	9.23%	G	C	0.977
ERBB4	8.60%	-	-	-	T	G	0.966
ERCC2	45.54%	46.97%	-	-	T	G	
ERCC3	7.86%	-	-	-	G	T	0.685
ERCC5	8.53%	-	-	-	G	A	0.734215
ERG	9.17%	-	-	-	C	A	0.015
EZH2	11.40%	-	-	-	C	T	0
FANCD2	35.00%	23.0%	30.44%	8.14%	-	-	
FBN1	12.12%	-	-	-	C	T	0.998
FBN2	-	-	-	24.49%	G	A	0.738
FBXW7	-	-	-	13.19%	G	A	1
FGFR1	-	-	-	10.70%	G	T	0.281
FGFR2	7.33%	-	-	-	C	T	0.08
FH	-	-	37.22%	-	C	G	0.012
FLT1	7.38%	-	-	20.69%			
FMN2	10.26%	-	-	-	C	T	0.6753
FZD3	7.77%	-	-	-	C	T	0.999
GNAQ	48.00%	40%	43%	28.00%	T	A	0.999
GSK3B	46.57%	50.90%	-	-	T	C	
IDH1	-	-	7.21%	-	G	C	0
IL7R	34.71%	54.97%	-	-	G	A	0.735
IMPA1	7.73%	-	-	-	C	T	0.273
INPP5B	7.86%	-	30.80%	-			
IRS2	-	7.89%	-	-	C	T	0.882
JAK3	-	-	-	7.14%	C	T	0.039
KAT6A	8.70%	-	-	-	G	T	0.755658
KAT6B	8.95%	-	-	-	C	T	0.735396
KDM1A	-	-	13.22%	-	C	T	0.058
KDM2A	8.06%	-	-	-	C	T	0.998
KDM3B	-	-	-	7.59%	G	A	
KDM5B	8.13%	-	-	-	C	A	0.998
KDM8	-	-	-	8.61%	C	T	1
KMT2A	-	-	-	9.02%	G	A	0
KMT2C	23.81%	9.26%	44.09%	8.09%	-	-	
KMT2D	16.44%	-	-	-	G	A	0
LRP5	13.04%	-	-	-	C	T	1
MAGI2	11.81%	-	-	-	C	A	0.194
MAP2K1	-	-	23.28%	-	C	T	0.384
MAP2K2	-	-	19.79%	-	C	T	0
MAP2K4	-	9.94%	-	-	C	A	0.063
MET	17.09%	-	-	-	G	T	0.999
MSH6	24.60%	27.40%	13.68%	6.46%	-	-	
NALCN	-	7.06%	-	-	C	A	1
NBN	37.98%	45.83%	-	-	G	A	0.657
NF1	15.71%	-	-	43.49%	C	G	0.989
NOTCH2	-	-	22%	-	G	A	0.709
NSD1	-	-	-	18.33%	G	A	0.995
NTRK2	-	-	10.93%	-	A	C	0.995
PBRM1	11.49%	-	-	-	G	A	0.793
PCDH15	-	22.64%	-	-	G	A	0.999
PIK3CA	38.20%	-	25.88%	-	G	A	
PIK3CB	7.35%	-	-	-	C	T	1
PIK3R3	25%	-	-	-	C	T	0
PIKFYVE	21.15%	-	-	-	G	T	0.999
PKM	-	-	7.90%	-	A	T	0.998
PMS1	8.98%	-	-	-	C	A	0.123
PPIP5K2	15.17%	10.29%	-	-	C	A	0.123
PRKCI	-	10.40%	-	-	G	A	0.435
RASA4	-	-	-	13.36%	C	T	0.007
RB1	14.81%	-	-	-	C	T	0.003
RICTOR	7.94%	-	-	-	G	T	0.796
SERPINB	12.55%	-	-	-	C	T	0.825
SETD2	-	-	-	40.32%	G	A	
SMAD2	-	11.22%	8.09%	-	G	A	0.513
SOX2	-	-	-	12.09%	G	A	0
SRC	-	-	99.81%	-	G	A	0.999
SYNE1	20.27%	9.52%	-	-	T	C	0.033
TGFBR1	-	7.01%	-	-	C	A	0.033
TSC2	17.36%	-	46.68%	-	G	C	0.958
WHSC1	-	-	-	48.65%	C	G	0.721
XIRP2	10.16%	-	-	-	G	A	0.24

Variant allele frequency, nucleotide in the allele variant and polyphen are shown. Genes mutated all samples are colored in gray
